# The soy-derived peptide Lunasin inhibits invasive potential of melanoma initiating cells

**DOI:** 10.18632/oncotarget.16066

**Published:** 2017-03-10

**Authors:** Chris Shidal, Jun-Ichi Inaba, Kavitha Yaddanapudi, Keith R. Davis

**Affiliations:** ^1^ Department of Pharmacology and Toxicology, University of Louisville School of Medicine, Louisville, Kentucky, USA; ^2^ James Graham Brown Cancer Center, University of Louisville School of Medicine, Louisville, Kentucky, USA; ^3^ Department of Biology and Biotechnology Program, Indiana University, Bloomington, Indiana, USA; ^4^ Department of Plant Pathology, University of Kentucky, Lexington, Kentucky, USA; ^5^ Department of Medicine, University of Louisville School of Medicine, Louisville, Kentucky, USA

**Keywords:** Lunasin, melanoma, cancer stem cells, integrin signaling, histone acetylation

## Abstract

Lunasin is a 44 amino acid peptide with multiple functional domains including an aspartic acid tail, an RGD domain, and a chromatin-binding helical domain. We recently showed that Lunasin induced a phenotype switch of cancer initiating cells (CIC) out of the stem compartment by inducing melanocyte-associated differentiation markers while simultaneously reducing stem-cell-associated transcription factors. In the present study, we advance the hypothesis that Lunasin can reduce pools of melanoma cells with stem cell-like properties, and demonstrate that Lunasin treatment effectively inhibits the invasive potential of CICs *in vitro* as well as *in vivo* in a mouse experimental metastasis model. Mice receiving Lunasin treatment had significantly reduced pulmonary colonization after injection of highly metastatic B16-F10 melanoma cells compared to mice in the control group. Mechanistic studies demonstrate that Lunasin reduced activating phosphorylations of the intracellular kinases FAK and AKT as well as reduced histone acetylation of lysine residues in H3 and H4 histones. Using peptides with mutated activity domains, we functionally demonstrated that the RGD domain is necessary for Lunasin uptake and its ability to inhibit oncosphere formation by CICs, thus confirming that Lunasin's ability to affect CICs is at least in part due to the suppression of integrin signaling. Our studies suggest that Lunasin represents a unique anticancer agent that could be developed to help prevent metastasis and patient relapse by reducing the activity of CICs which are known to be resistant to current chemotherapies.

## INTRODUCTION

Melanoma is a notoriously aggressive form of skin cancer that represents approximately 80% of all skin cancer related deaths, despite accounting for only 5% of diagnosed cases [1, 2]. New classes of small-molecule inhibitors combating malignant melanomas have yielded mixed results [3–5]. Although many patients achieve an initial tumor regression, these agents quickly become ineffective, and additionally, may promote the spread of a highly aggressive and chemoresistant population of cells [6–8]. Studies utilizing immunotherapy (extensively reviewed in [9]) to treat malignant melanomas have been found to be an effective treatment option; however, only a relatively small subset of patients achieve a sustained complete response [10–12]. More recently, immunotherapies with substantially improved objective responses in melanoma patients have supported the clinical utility of immunotherapy [13–16]. Nevertheless, adverse safety profiles, chemoresistance, and immune evasion continue to prove problematic in many of these newly approved immunotherapies [17–19]. Thus, providing patients with additional novel adjuvant therapies to reduce or even prevent metastatic spread will continue to be needed for the development of effective treatment strategies that result in long-term survival.

The process of invasion and metastasis is perhaps the most significantly studied hallmark of cancer due to the high mortality rates caused by the metastatic dissemination of tumor cells from the primary tumor into distant organs. Malignant melanoma metastases can frequently be found in the brain, lymph nodes, gastrointestinal tract, liver, and most commonly the lungs [20]. Primary tumor formation and subsequent metastatic outgrowth is maintained by a subset of cells with innate stem cell-like abilities that enable them to invade and colonize surrounding tissues, while preserving a population of highly proliferative bulk tumor cells [21, 22]. The heterogeneous nature of melanomas make an intriguing model to study metastatic dissemination as they have been reported, among many classes of solid tumors, to harbor cancer initiating cell (CIC) populations identified by several biomarkers including aldehyde dehydrogenase (ALDH) [23, 24], CD20 [21], CD133 [25], CD271 [26], and ABCB5 [22].

**Figure 1:**
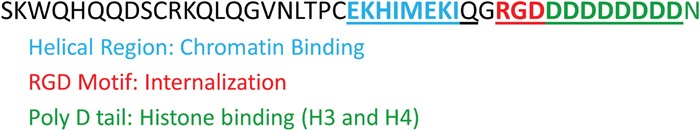
Amino acid sequence of the Lunasin peptide Lunasin is a 44 amino acid peptide with 3 functional domains attributed with its anticancer activity: 1) a helical regional conserved in chromatin-binding proteins (blue), 2) a RGD motif recognized by integrins (red), and 3) a poly-aspartic acid tail involved in histone-tail binding (green).

Lunasin is a 44 amino acid peptide [27, 28] isolated from soy that has been shown to have chemopreventive and chemotherapeutic activity [29–37]. Lunasin has three domains implicated in its anticancer activity; an RGD motif, a helical domain with a sequence conserved in chromatin binding proteins, and a poly-aspartic acid tail (Figure 1). We recently reported that Lunasin significantly reduced a putative melanoma stem cell population expressing elevated levels of ALDH [37]. Additionally, we showed that *in vivo* tumor growth initiated by this putative CIC population was significantly impaired in mice treated with Lunasin. Previously, Lunasin was shown to inhibit metastasis of malignant colon cancer cells and additionally, potentiated the antimetastatic effects of oxaliplatin [38]; however, studies linking Lunasin to suppressed metastatic dissemination are largely lacking. With the encouraging effects of Lunasin on breast and melanoma CICs [37, 39], it is plausible to speculate that by reducing expansion of the CIC compartment, Lunasin would ultimately decrease the ability of tumor cells to invade, survive, and colonize distant tissues.

Preliminary studies of Lunasin suggested that a primary anticancer mechanism was derived from its activity as a HAT inhibitor [29]. Both HAT inhibitors and their counter, histone deacetylase (HDAC) inhibitors, have been shown to have potential clinical utility in malignant melanoma [40, 41]; however, these agents may also contribute to undesirable effects. For example, it was recently published the HDAC inhibitor, valproic acid, caused breast cancer cells to dedifferentiate toward a chemoresistant stem-like state [42]. With regard to Lunasin, we found that histone acetylation patterns are altered in non-small cell lung cancer (NSCLC) and melanoma; however, it is an open question as to whether or not it is a driving mechanism in Lunasin's chemotherapeutic activity. Our previous studies suggest that inhibition of integrin signaling is a primary mechanism that causes the effects seen in NSCLC models [34, 43]. The relationship of changes in histone acetylation and integrin signal transduction remains unclear. One major question that remains to be answered is whether integrin signaling can modulate epigenetic histone modifications or vice versa?

Two key signaling pathways involved in the metastatic cascade are the integrin-FAK axis [44] and the downstream PI3K/AKT pathway [45]. FAK is a critical mediator of cell proliferation, differentiation, angiogenesis, and invasion as it promotes cytoskeletal remodeling through interactions with several proteins including Src kinases [46]. The PI3K/AKT pathway is also found to be aberrantly regulated in a variety of cancers including melanoma [47]. Although generally thought of as a central protein involved in cell survival and cell cycling, AKT has been shown to bind and regulate FAK phosphorylation suggesting an important role in metastatic adhesion [48]. Dual targeting of these dysregulated pathways by disrupting upstream (integrin) signaling remains a promising therapeutic approach despite the fact that there are few clinical applications using this approach. Pharmacologic targeting of integrins is currently undergoing clinical trials for the treatment of malignant melanomas [49]. Due to the central role of integrins in several oncogenic signaling pathways [50], blockade of integrin signal transduction seems a likely candidate for future drug development. While the potential clinical benefit of integrin antagonists remains promising, this class of drug will likely be utilized in combination with more traditional chemotherapeutics [51–54].

The present study significantly extends our previous work by demonstrating that Lunasin inhibits metastasis-associated activities in melanoma CICs both *in vitro* and *in vivo* and supports the notion that this multifaceted peptide with a complementary array of mechanisms has the potential to be used as an adjuvant therapy against malignant melanomas compared to single-agent treatment strategies.

## RESULTS

### Lunasin uptake correlates with expression of α_V_ integrin subunits

**Figure 2:**
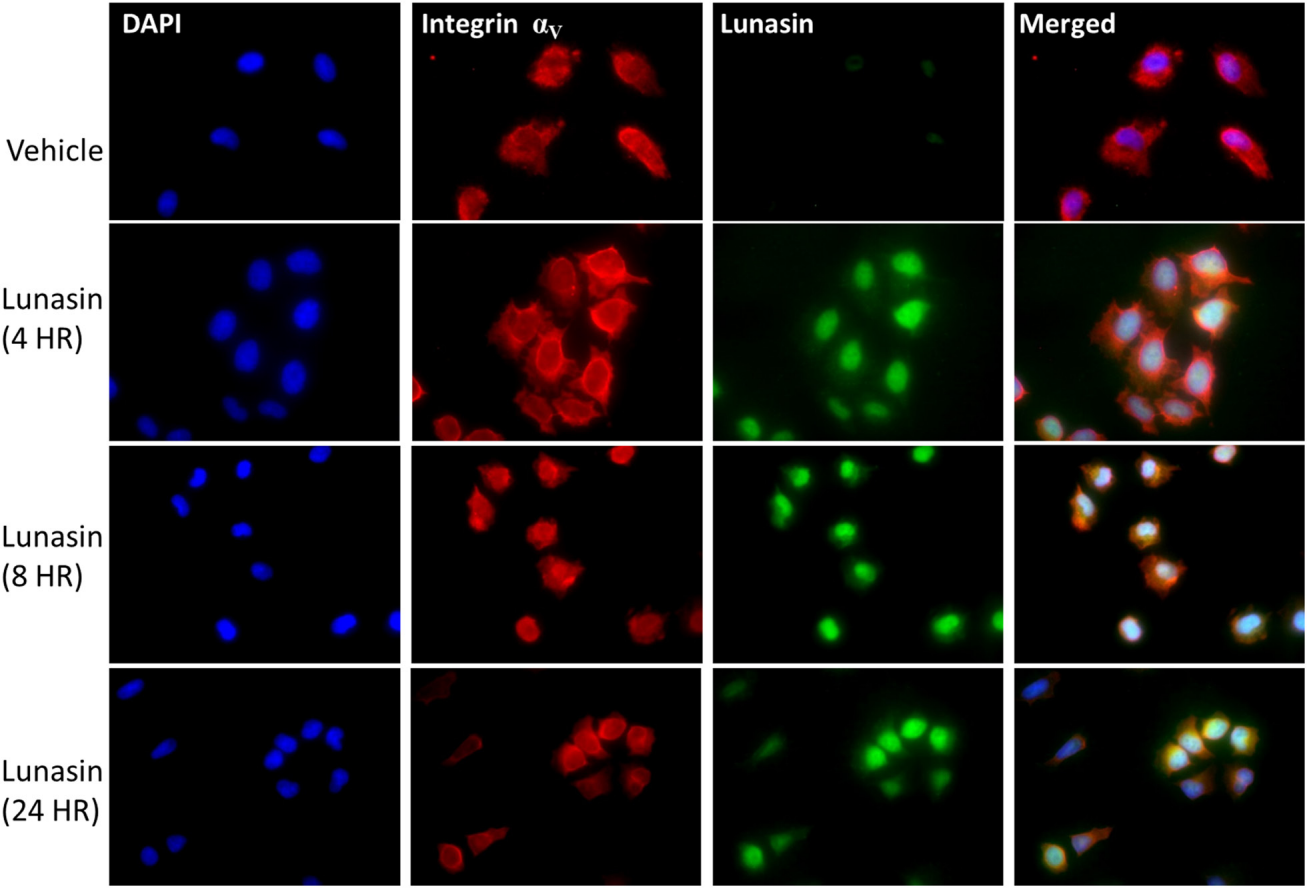
Lunasin is readily internalized by A375 melanoma cells A375 cells treated with Lunasin for up to 24 h internalized Lunasin, which was found to colocalize with integrin α_V_ subunits. Additionally, we observed nuclear localization of Lunasin that persisted after 24 h of treatment. We also observed the clustering of integrin subunits around the nucleus when A375 cells were treated with Lunasin suggesting the endocytic mechanism for Lunasin internalization involved integrins. Representative images from three independent experiments were used, and were taken at 40x magnification. (Blue = dapi, green = Lunasin, red = integrin α_V_).

Lunasin internalization is thought to be dependent upon endocytic mechanisms involving integrins [55]. A375 cells, which overexpress the integrin α_V_β_3_, were treated with vehicle or 100 μM Lunasin, and analyzed for co-localization of integrin subunits and Lunasin at several time points ranging from 4 to 24 hours. Lunasin was rapidly internalized in A375 cells and was present both in the cytoplasm and the nucleus. Interestingly, cell morphology was also altered at later time points in Lunasin-treated cells; a decrease in cell size as well as localization of integrins around the nucleus was observed in our treated cells when compared to controls (Figure 2). Although Lunasin has been shown to have antiproliferative effects in NSCLC, we did not observe a statistically significant effect on cell cycle or cell viability, albeit, we did observe a modest increase in the G_1_ population; when A375 and B16-F10 cells were treated with Lunasin (Supplementary Figure 1).

### B16-F10 CIC populations were reduced with Lunasin treatment

**Figure 3:**
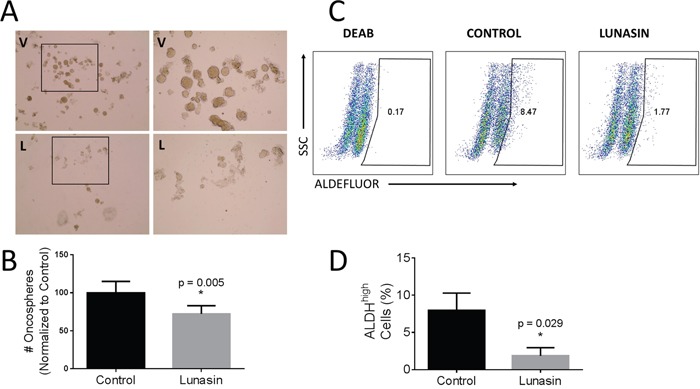
Lunasin disrupted oncosphere formation and reduced ALDH^high^ populations B16-F10 ALDH^high^ cells were plated in low adherent culture in stem cell media and allowed to form floating oncospheres. When media was amended with 100 μM Lunasin, we observed a significant decrease in oncosphere formation compared to control samples **(A, B)**. V = vehicle, L = Lunasin. ALDH activity was measured as previously described. When B16-F10 cells were treated with Lunasin, we observed a significant reduction in cells displaying the ALDH^high^ phenotype **(C, D)**. Figures represent data obtained from three independent experiments and are shown as mean ± s.d. Statistical significance (*p* < 0.05) was determined by student's t-test and denoted by an asterisk (*).

Previously, we showed Lunasin reduced ALDH-expressing populations of cells in A375 and SK-MEL-28 melanoma cell lines concomitant with a decreased ability of these cells to form oncospheres when plated in anchorage-independent culture conditions in serum-free media [37]. To determine if this is the case with a murine model of melanoma, we repeated the experiments using the aggressive mouse-derived B16-F10 melanoma line. Representative images taken at 7 days post-treatment show the inhibitory effect of Lunasin on oncosphere formation (Figure 3A). Treatment with 100 μM Lunasin reduced oncosphere formation by 29% (*p* = 0.005, Figure 3B). Additionally, we observed a decrease in the ALDH^high^ population when cells were treated with 100 μM Lunasin for 24 h (Figure 3C and 3D). Treatment reduced the mean percentage of ALDH-positive B16-F10 cells from 8% in the control samples to 1.9% in the Lunasin-treated samples (*p* = 0.029).

### Lunasin inhibits invasion of ALDH^high^ melanoma stem cells

**Figure 4:**
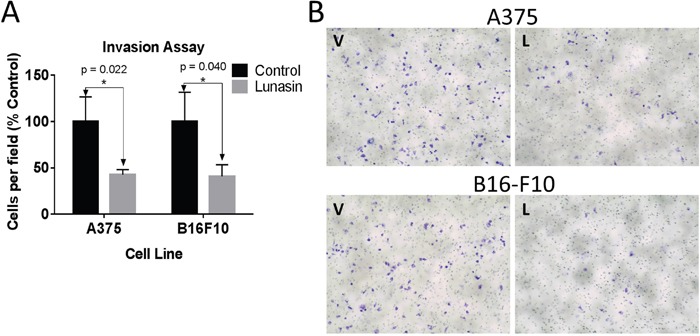
CIC invasion was suppressed in Lunasin-treated cells *In vitro* invasion assays demonstrate that Lunasin-treated A375 and B16-F10 ALDH^high^ cells had less invasive capacity than cells treated with vehicle **(A)**. Invading cells were stained with toluidine blue and representative images are shown at 20x magnification **(B)**. Data from three independent experiments are shown as mean ± s.d. Statistical significance was determined by student's t-test and denoted by an asterisk.

A375 and B16-F10 cells were sorted to isolate populations with elevated ALDH activity. These cells were pretreated with 100 μM Lunasin for 24 h, and subsequently replated in the upper chamber of transwell inserts containing serum-free DMEM/F12 media amended with PB or Lunasin. After adding media containing 10% FBS to the lower chamber, plates were incubated for 24 h, and the cells invading through the Matrigel basement membrane were counted. Invasion of A375 and B16-F10 ALDH^high^ cells was significantly inhibited in Lunasin-treated wells compared to vehicle-treated wells resulting in a 57% (*p* = 0.02) and 60% (*p =* 0.04) decrease in invading cells, respectively (Figure 4A). Representative images showing the Toluidine-stained invading cells from the bottom of the inserts illustrate the anti-migratory effects of Lunasin *in vitro* (Figure 4B).

### Lunasin abrogates pulmonary metastasis *in vivo*

**Figure 5:**
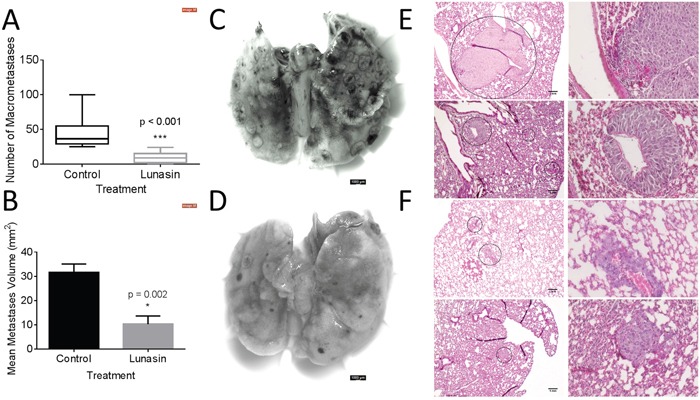
Lunasin reduced pulmonary metastases *in vivo* B16-F10 melanoma cells were injected IV into female C57BL/6 mice. Lunasin-treated mice had less incidence of macrometastases **(A)** as well as significantly reduced average lesion area **(B)**. Representative images of pulmonary tissues resected from control **(C)** and Lunasin **(D)** treated mice are shown. H&E stained sections demonstrate the significant difference between average lesion area in vehicle **(E)** and Lunasin **(F)** treated mice. Stained sections were imaged at 10x (left) and 40x (right), scale bar = 1 mm. Graphs represent data plotted as mean ± s.e.m. Means were determined to be statistical significant by student's t-test and significance is denoted by an asterisk.

To test whether Lunasin's antimetastatic effects would persist *in vivo*, we employed a syngeneic mouse model using the B16-F10 cell line. We previously validated this system as a model to test Lunasin's efficacy in inhibiting tumor growth [56]. When 2.5×10^5^ B16-F10 cells were intravenously injected into C57Bl/6 mice, pulmonary seeding and subsequent tumor establishment occurred within 18 days. Throughout the experiment, mice were dosed daily with vehicle or Lunasin (30 mg/kg) by intraperitoneal (IP) injection. Upon experimental endpoint, we observed that Lunasin-treated mice had significantly reduced metastatic outgrowths when compared to control mice (Figure 5). Mice in the control group averaged 45 (± 22) observable pulmonary lesions compared to only 9.5 (± 8) in Lunasin-treated mice (Figure 5A). Representative images of lungs resected from metastasis-bearing mice in control (Figure 5C) and Lunasin (Figure 5D) treatment groups are shown. In addition, macrometastases were present in the lungs of all control mice (n = 10); however, lungs from 2 mice in the Lunasin group (n = 10) had no observable macrometastases (Supplementary Figure 2).

Hematoxylin and eosin (H&E) stained lung sections also showed an observable difference in the average area of micrometastases between treatment groups. Control mice had an average lesion area of 31.6 mm^2^ compared to 10.3 mm^2^ in the Lunasin group (Figure 5B). Micrometastases formed in the lungs of vehicle treated mice (Figure 5E) were larger and more abundant than in mice treated with Lunasin (Figure 5F). Cellular morphology was similar in lesions found in the lungs of both control and Lunasin-treated mice.

### Lunasin antagonizes integrin signaling through FAK/AKT/ERK and inhibits histone acetylation

**Figure 6:**
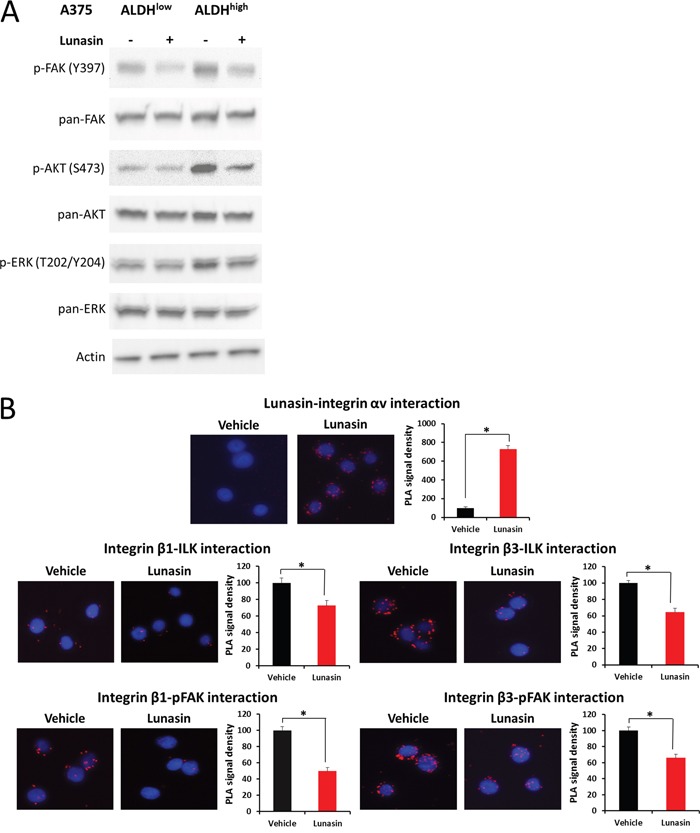
Lunasin suppressed integrin signal transduction ALDH^high^ and ALDH^low^ cells derived from the A375 melanoma cell line were treated with 100 μM Lunasin in low adherent culture for 24 h, and the resulting lysates were probed for integrin-associated signaling proteins **(A)**. We observed a significant difference in phosphorylation patterns of FAK, AKT, and ERK in ALDH^high^ cells, while only a modest effect was observed in ALDH^low^ cells **(A)**. Actin was used as a reference protein. Additionally, we used PLA assays to validate that Lunasin was targeting integrin signal transduction. Our results suggest Lunasin decreased the interactions between integrin β subunits and the intracellular kinases FAK and ILK **(B)**. Furthermore, we show that Lunasin specifically interacts with the RGD-recognizing α_V_ integrin subunit **(B)**.

We next investigated whether the effects of Lunasin on human and murine melanoma cells is related to the known effects of Lunasin on integrin signaling and histone acetylation. Immunoblot analysis showed that A375 and B16-F10 melanoma lines treated with Lunasin for 24 h had decreased phosphorylation of FAK, AKT, and ERK. When compared to ALDH^low^ cells, A375 ALDH^high^ cells showed an increased sensitivity to Lunasin's disruption of AKT and ERK phosphorylation (Figure 6A); both ALDH^low^ and ALDH^high^ showed decreased FAK phosphorylation. Interestingly, ALDH^high^ and ALDH^low^ A375 cells had somewhat contrasting integrin expression profiles; ALDH^high^ cells expressed integrin subunits α_V_ and β_3_ more abundantly than ALDH^low^ cells when grown in anchorage independent culture, while ALDH^low^ cells seemed to express higher levels of α_5_ and β_1_ integrin subunits (Supplementary Figure 3).

In order to extend and validate the effects of Lunasin on integrin signaling in CICs, we utilized proximity ligation assays (PLA) to investigate the interactions between integrin β subunits and the intracellular signal transducers ILK and pFAK (Y397). Initially, we observed that Lunasin interacts with the integrin α_V_ subunit of A375 ALDH^high^ cells (Figure 6B). This interaction suppressed downstream interactions between β_1_ and β_3_ integrin subunits with ILK and pFAK by approximately 45% (Figure 6B). These results are consistent with those seen in our NSCLC models [34], and further confirm that the effects of Lunasin on melanoma CICs are, in part, due to altered integrin signaling pathways.

**Figure 7:**
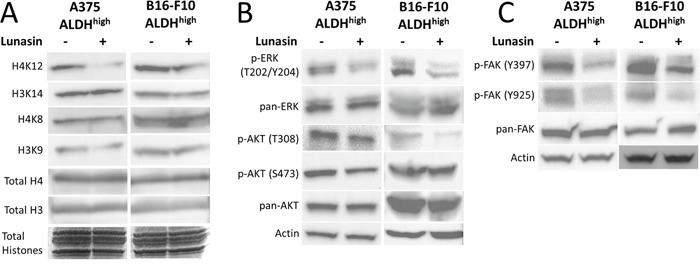
Lunasin inhibited phosphorylation of FAK, AKT, and ERK as well as histone acetylation ALDH^high^ cells derived from human A375 and murine B16-F10 melanomas were treated with Lunasin for 24 h, and the resulting cell lysates were subjected to SDS-PAGE and probed for integrin-associated signaling pathways. We observed a decrease in histone acetylation at H3K9 and H4K12 **(A)**, which suggests epigenetic modification may play a role in Lunasin's effects on melanoma CICs. As previously shown, these changes are concomitant with inhibition of activating phosphorylations of AKT **(B)**, ERK **(B)**, and FAK **(C)**. Actin was used as a reference protein for all immunoblot analysis.

Because Lunasin has been reported to modulate histone acetylation, we also tested if any histone acetylation marks may have been altered by Lunasin treatment. Histones were isolated by acid extraction, and acetylation marks in H3 and H4 histone were assessed by immunoblot analysis. Previously, we identified several acetylation marks were altered when NSCLC cells were incubated with Lunasin [34]. Interestingly, we observed a change in a different set of acetylation marks in melanoma cells treated with Lunasin. Lunasin treatment reduced histone acetylation at H3K9 and H4K12, while no difference was seen in acetylation of H4K8 and H3K14 (Figure 7A). These data indicate that Lunasin indeed modulates HAT activity in melanoma cells resulting in decreased acetylation marks, which may contribute to the anticancer effects of Lunasin. These changes in histone acetylation were concomitant with reduced FAK, AKT and ERK1/2 phosphorylation in Lunasin-treated A375 and B16-F10 ALDH^high^ cells. Lunasin treatment decreased phosphorylation of AKT at S473 and T308 as well as phosphorylated ERK1/2 at T202/Y204 (Figure 7B). We also observed that phosphorylation of FAK at tyrosine residues Y397 and Y925 were inhibited when ALDH^high^ cells were treated with Lunasin for 24 h (Figure 7C). These results confirmed our initial data indicating that Lunasin significantly disrupts integrin signal transduction through altering phosphorylation patterns of several key proteins (Figure 6).

### The RGD-domain is essential for Lunasin uptake and disrupting oncosphere formation

**Figure 8:**
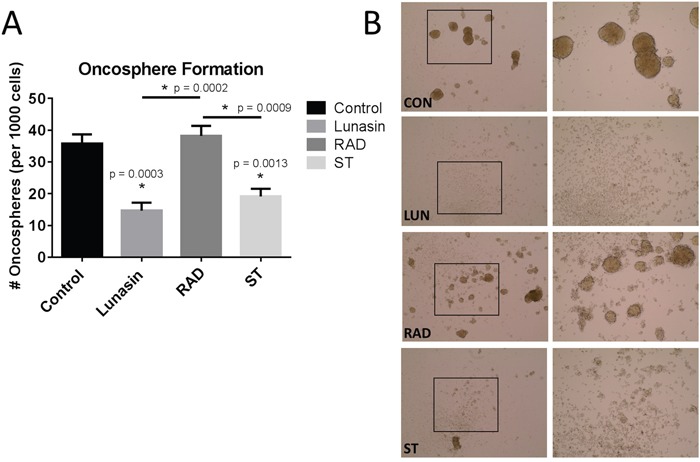
Lunasin's RGD motif is essential for disrupting oncosphere formation Mutated peptides with alterations in the RGD domain and the poly-aspartic-acid tail were used to treat A375 ALDH^high^ cells in low adherent culture. Vehicle-treated cells readily formed oncospheres, but native Lunasin disrupted oncosphere formation **(A)**. When the RGD sequence was mutated to RAD, Lunasin lost its ability to inhibit oncosphere formation, while a peptide containing a scrambled tail retained the ability to inhibit oncosphere generation **(A)**. Representative images taken at 10x (left) and 20x (right) demonstrate the ability of the peptide to inhibit oncosphere formation **(B)**. Averages from three independent experiments were plotted as mean ± s.d. Statistical significance was determined by student's t-test and denoted by an asterisk.

Our immunoblot and PLA analyses implicated suppression of integrin signaling and effects on histone acetylation as potentially being important for Lunasin action. To further investigate whether these mechanisms are required for Lunasin activity, we synthesized peptides in which the RGD domain or poly-aspartic acid tail were mutated in order to disrupt Lunasin's interaction with integrins or core histones, respectively. We used the formation of oncospheres as a surrogate assay to identify the effect of Lunasin on CIC clonogenicity. We observed no difference in the ability of A375 ALDH^high^ cells to form oncospheres compared to vehicle-treated cells and cells treated with RAD-mutated peptide (Figure 8). Conversely, cells treated with native Lunasin (*p* < 0.001) and the scrambled tail peptide (*p =* 0.0013) had a significantly reduced ability to form oncospheres in anchorage-independent culture (Figure 8A and 8B). These data suggest that the RGD domain, which interacts with integrins, is necessary for preventing sphere formation by CICs whereas the poly-aspartic acid tail is not.

**Figure 9:**
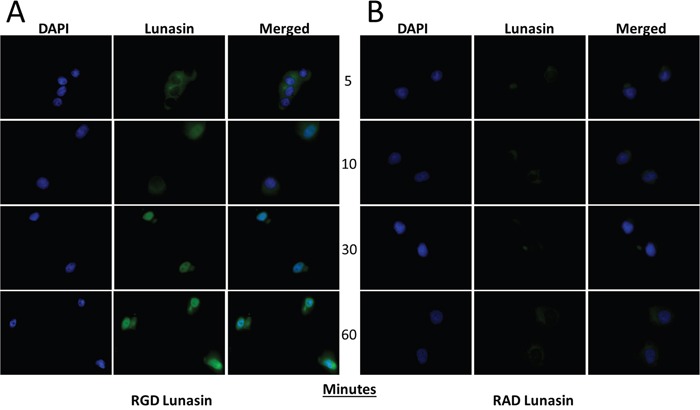
Lunasin uptake is an integrin-dependent process RGD **(A)** and RAD **(B)** Lunasin peptides were incubated with A375 cells for various time points up to 1 h. While some RAD-Lunasin was detected, cells treated with RGD-Lunasin (native) showed significantly more abundant intracellular localization as illustrated by a significantly increased fluorescent signal. Additionally, we observed that native Lunasin (i.e. RGD) was localized in the nucleus of A375 cells after just 10 minutes, while RAD-Lunasin was detected mainly in the cytoplasm. Images are representative of data obtained from two independent experiments, and were analyzed using ImageJ software.

An endocytic mechanism by which Lunasin is internalized has been reported in human macrophages [55]. Since the RGD domain appears to be necessary for Lunasin's disruption of oncosphere formation, we next questioned whether or not the RGD domain was essential for Lunasin's internalization. A375 cells, which express the RGD-recognizing integrin subunits α_V_ and α_5_ (data not shown), were treated with 100 μM of native Lunasin (Figure 9A) or RAD-Lunasin (Figure 9B) for 5, 10, 30, and 60 minutes. Cells were fixed and probed for Lunasin using a rabbit polyclonal antibody which was confirmed to recognize the mutated peptide sequence (Supplementary Figure 4). Although Lunasin was detected intracellularly in cells treated with both native and RAD-mutated peptides, fluorescent intensity was much higher in cells treated with native Lunasin compared to RAD-Lunasin. Interestingly, RAD-Lunasin never localized in the nucleus at detectable levels, while the native peptide was observed in the nucleus after 10-30 minutes. These data support the notion that Lunasin's internalization is largely integrin-dependent.

## DISCUSSION

Our findings that Lunasin reduced the metastatic potential of melanoma CICs both *in vitro* and *in vivo* support the earlier indications that RGD peptides may help alleviate patient relapse in malignant melanomas. We show that mechanisms previously described for Lunasin's anticancer effects persist in our melanoma models, and perhaps most importantly, are exacerbated in isolated CIC populations. We showed that uptake of Lunasin in A375 cells was integrin-dependent and correlated with the expression of the integrin α_V_ subunit. Colocalization of Lunasin with integrin subunits was observed at several time points varying from 4 h to 24 h, and localization of Lunasin in both the cytoplasm and nucleus was observed for all time points. These data are in agreement with previously reported studies on Lunasin's interaction and uptake with specific integrin subunits [55, 57]. Interestingly, we observed a morphological difference between A375 cells treated with Lunasin and vehicle. Intracellular localization of integrin α_V_ in Lunasin-treated cells was observed, while integrin α_V_ was found only on the periphery of vehicle-treated cells. These data indicate that Lunasin was readily internalized in A375 cells, and support the previously described endocytic mechanism reported in human macrophages [55].

We previously published results using human melanoma cell lines showing that Lunasin efficiently reduced pools of CICs based on the ALDH biomarker, and resulted in disrupted oncosphere formation when ALDH^high^ cells were plated in stem cell media in anchorage-independent culture conditions [37]. Additionally, we found that Lunasin induced expression of the melanocyte-associated differentiation markers MITF and Tyrosinase. Low-MITF expressing populations in melanomas have been described to harbor a slow cycling stem-like population with intrinsic chemoresistant and tumorigenic properties [58]. It was recently reported that MITF regulates melanoma invasion through Rac/Rho GTPases [59], which supports previous evidence showing MITF is explicitly involved in melanoma progression [60, 61]. This is a particularly interesting discovery given the regulation of Rac1 by integrins [62].

In our present studies, we showed a significant decrease in B16-F10 oncosphere generation when treated with 100 μM Lunasin concomitant with a significant decrease in the ALDH-positive population of cells, which has been reported to include the CIC fraction responsible for tumor formation and metastasis [24]. When the *in vitro* invasive potential of A375 and B16-F10 ALDH^high^ cells was measured, Lunasin-treated cells were significantly less capable of invading through an artificial basement membrane when compared to vehicle-treated cells. These data are in agreement with Lunasin's effect on depleting ALDH^high^ populations [37], which may be responsible for metastatic dissemination [23, 24, 37].

When C57Bl/6 mice were subjected to an experimental metastasis model of melanoma using B16-F10 cells, Lunasin treatment significantly suppressed the ability of these cells to invade and proliferate in the lungs. Several mice in the Lunasin-treated group displayed no sign of macrometastases suggesting that Lunasin was an effective treatment for reducing or abolishing metastatic burden altogether. We previously demonstrated that Lunasin inhibited subcutaneous tumor growth of murine models of melanoma and NSCLC [56]. Utilizing immunocompetent preclinical models of cancer allows researchers to explore the complex relationship between host immunity and tumor microenvironment; this especially holds true given the immunogenic nature of melanomas [63, 64]. It has been found that Lunasin may have immune boosting effects, and may improve vaccine efficacy by promoting dendritic cell maturation [65, 66]. Furthermore, Lunasin synergistically enhanced the cytotoxic effect of NK cells when combined with cytokine therapy [66]. The exciting possibility that Lunasin not only directly affects cells by reducing integrin signaling or histone acetylation, but can also “prime” the innate immune system to repress cancer cell proliferation illustrates the extremely promising benefits of this peptide that deserve further study.

As previously described [37], Lunasin has a selective effect on melanoma CICs compared to bulk tumor cells. We wanted to determine whether these selective effects would persist when integrin signal transduction was evaluated. Phosphorylations of FAK, AKT, and ERK, intracellular kinases downstream of integrins, were significantly reduced when A375 and B16-F10 cells were treated with Lunasin; A more robust effect was observed in the ALDH^high^ cells when compared to the ALDH^low^ cells. These mechanisms have been described in several cancer models including breast [36], colon [38], and lung cancer [34]; however, this is the first report that CICs are more sensitive to Lunasin's integrin antagonism in melanoma. Given the explicit involvement of FAK and AKT in carcinogenesis, progression, and metastasis [44, 45], our results are promising especially taken in conjunction with our finding that Lunasin decreased CIC pools.

The disparate Lunasin sensitivity of A375 ALDH^high^ and ALDH^low^ cells correlates with differential expression of specific integrin subunits when cultured as spheres in low-adherent conditions. ALDH^high^ cells expressed relatively higher amounts of α_V_ and β_3_ integrin subunits, while ALDH^low^ cells expressed comparatively higher levels of α_5_ and β_1_ integrin subunits. The complexities of integrin signal transduction mechanisms remain somewhat of a mystery, however; new research has revealed differences in signaling coordinated though α_v_β_3_ integrins and α_5_β_1_ [67, 68]. In fact, expression of specific integrins in stem cell-like cancer cells has been reported in other cancer models including prostate [69] and breast [70], which may reveal a potential weakness of CICs that provides a potential therapeutic target to specifically inhibit CIC expansion.

Histone acetylation by HATs (Reviewed in [71]) results in the unwinding of chromatin from the nucleosome complex consisting of H3 and H4 histones; efficient histone acetylation is necessary for the initiation of transcription of target genes. Thus, targeting HATs appears an attractive means to reduce cancer cell proliferation. Lunasin's activity as a HAT inhibitor has been described [72]. Histone acetylation in A375 and B16F10 ALDH^high^ cells was affected with Lunasin treatment; however, Lunasin induced different acetylation patterns in melanoma when compared to our previously reported results in NSCLC [34]. Recently, it was shown that HAT inhibition preferentially induced apoptosis and inhibited stem-associated markers in a NSCLC model [73]. While we do not see an induction of apoptosis in our melanoma models, our results parallel those obtained in this study, suggesting a potential link between suppression of CIC invasion and Lunasin's epigenetic mechanisms.

Our studies suggest that inhibition of integrin signaling is the primary mechanism mediating Lunasin's effects in melanoma stem cells. When the RGD domain of Lunasin was mutated, Lunasin lost its ability to disrupt oncosphere formation, a surrogate assay for stem cell identification and propagation. Mutating the poly-aspartic acid tail seemingly had no effect on oncosphere formation; this implies that effects on histone acetylation mediated by the poly-aspartic acid tail are not required for inhibiting CIC clonogenicity. Though supporting evidence shows that stem cells can be maintained through integrin signaling [74], further research must be conducted to unequivocally determine that integrin antagonism is the sole mechanism for Lunasin's reduction of the CIC compartment, especially given that CICs may also be maintained by histone acetylation [75]. This is particularly interesting considering that when p300, a HAT whose activity is antagonized by Lunasin [76], was knocked out in embryonic stem (ES) cells, Nanog expression was markedly reduced; however, self-renewal capacity (a function measured by oncosphere formation) was not significantly affected [77]. These results corroborate findings from our previous study [37] showing Lunasin treatment resulted in a significant reduction in Nanog levels, however, Lunasin treatment also robustly inhibited sphere formation, suggesting oncosphere formation may be integrin-dependent and independent of histone acetylation. Crosstalk between integrin signaling and histone acetylation is relatively unexplored, however, evidence that integrin β_1_ engagement with ECM proteins may regulate H3 acetylation patterns has been described [78]. The complex signaling circuits between extracellular cues transduced through integrins, and intracellular events leading to changes in histone acetylation patterns is slowly unraveling; however, several key pieces of the puzzle remain to be identified. Lunasin may serve as a key tool to bridge the gap between these two interesting and highly complex signaling pathways.

In summary, the present study found that Lunasin has robust antimetastatic properties *in vitro* and *in vivo*. CICs characterized by elevated ALDH activity showed greater sensitivity to Lunasin's antagonism of integrin signaling, as assessed by downstream activating phosphorylations of FAK and AKT. In agreement with our initial studies, we showed that B16-F10 cells exhibited Lunasin-dependent depletion of ALDH^high^ populations, and disruption of oncosphere formation. While Lunasin also altered histone acetylation patterns, Lunasin's effects in melanoma appear to be largely an integrin-dependent process. We extended our earlier work showing that using Lunasin to reduce pools of CICs may provide a new strategy to decrease invasion and subsequent metastatic outgrowths from this metastatic cell population. By modulating integrin signaling through FAK and PI3K/AKT pathways as well as altering histone acetylation patterns, Lunasin's complex and multifaceted anticancer activities suggest a potential therapeutic utility against malignant diseases in which recurrence due to CICs is likely. Given our results as well as those from others, a sufficient body of evidence has been obtained to justify further examination of the clinical utility of Lunasin as an anti-metastatic agent in patients with late stage cancers that are at risk of further metastatic dissemination.

## MATERIALS AND METHODS

### Purification of Lunasin from defatted soy and synthesis of mutated peptides

Lunasin was isolated from defatted soy flour as previously described [27]. Mutated peptides were synthesized by China Peptides (Shanghai, China) with purity > 95% as assessed by HPLC/MS. Full sequences are provided (Supplementary Figure 5). Peptides were dissolved in 50 mM sodium phosphate buffer pH = 7.4 (PB) and 0.5 ml aliquots were placed into 2 kD molecular weight cutoff Slide-a-Lyzer cassettes (Thermo-Fisher). Cassettes were dialyzed against 2 liters of PB, with the dialysis buffer being refreshed three times over the 24 h dialysis period. Protein concentrations from the resulting dialyzed peptide solutions were determined using a bicinchoninic acid (BCA) assay (Pierce) following the manufacturer's instructions for the microplate procedure. Purified bovine serum albumin (Pierce) was used as a standard. Peptide solutions were filter sterilized by passing through a 0.22 μm filter (Millipore), aliquoted, and stored at -20°C until use.

### Cell culture and reagents

A375 and B16-F10 cells were obtained from the American Type Culture Collection (Rockville, MD). Cells were analyzed for mycoplasma contamination every 6 months. Both cell lines were maintained in Dulbecco's Modified Eagles Medium (DMEM) supplemented with 10% Fetal Bovine Serum (FBS), Penicillin (100 U/mL), and Streptomycin (100 μg/mL). Cells were incubated at 37°C at 5% CO_2_ and sub-cultured every 72 hours. ALDH^high^ cells were isolated by fluorescence-activated cell sorting (FACS) [37] and were grown in DMEM/F-12 serum-free media (SFM) containing 1 × N-2 Supplement (Gibco) 10 ng/mL basic fibroblast growth factor (Gibco), and 10 ng/mL epidermal growth factor (Gibco). All experiments were done with cells that had been sub-cultured from 2 to 15 passages after the initial passage following removal from cryostorage.

### Immunofluorescence

A375 cells were plated in DMEM culture media at a density of 1 × 10^4^ cells per well in an 8-chambered microscope slide. Cells were allowed to adhere for 4 hours before removal of media and replacement with media containing vehicle (PB) or 100 μM Lunasin. Cells were allowed to incubate with treatment media for up to 24 h. At selected times, cells were washed with PBS, fixed with 4% paraformaldehyde, and permeabilized with 0.1% Triton X-100. Cells were incubated at -20°C in 100% methanol before blocking with 1% bovine serum albumin. Cells were incubated with anti-Lunasin (1:1000) rabbit polyclonal antibody and anti-α_V_ (1:100) mouse monoclonal antibody (Santa Cruz #376156) in blocking solution. Following overnight incubation, cells were washed and incubated with appropriate secondary antibodies conjugated to AlexaFluor-488 or AlexaFluor-647 fluorophores (Jackson ImmunoResearch). After washing, mounting media containing DAPI (Thermo Fisher) was dropped onto slides, and the slides were sealed using a 60 mm cover slip and clear finger nail polish prior to fluorescent analysis. Images were taken on a Nikon NiE upright microscope using Nikon Elements software (Nikon).

### Proximity ligation assay (PLA)

A375 ALDH^high^ cells were isolated by FACS as described elsewhere [37]. Briefly, cells were washed twice with PBS, plated on glass coverslips coated with poly-lysine (Sigma), and treated with 100 μM of Lunasin for 24 h. After treatment, coverslips were washed and probed with appropriate antibodies against Lunasin, phosphorylated focal adhesion kinase (FAK), integrin-linked kinase (ILK), and integrin α_V_ and β_1_ subunits as previously described [34]. Antibodies were then labelled using the Duolink *in situ* red starter kit (Sigma) following the recommended manufacturer's protocol, and subsequently imaged on a Nikon NiE upright microscope with Nikon Elements software (Nikon). Fluorescence analysis of at least 40 cells per sample were analyzed using ImageJ software (NIH).

### Oncosphere formation assay

A375 and B16-F10 CICs were isolated based on ALDH activity as previously described [37]. Gates for each sample were based upon N, N-diethylaminobenzaldehyde (DEAB) controls for each cell line. Sorted cells were cultured in low-adherent 6-well plates (Corning) in serum-free media at a density of 1 × 10^3^cells/mL. Cultures were grown for up to 14 days and treated with fresh media containing either 100 μM Lunasin or vehicle (PB) twice per week. Oncospheres (> 100 μm) were counted and imaged using an EVOS light microscope (Life Technologies) and images were analyzed using Image-J software (NIH) as described [37].

### Flow cytometry

ALDH activity was evaluated using the ALDEFLUOR^TM^ (StemCell Technologies) staining kit as described [37]. Cells were treated with vehicle or Lunasin for 24 h, harvested, and subjected to ALDEFLUOR staining. The 24 h treatment period was selected based on our previously published studies of Lunasin treatment of melanoma [37] and NSCLC [34, 35] cells where we observed significant effects on cell cycle and integrin signaling. The data were analyzed using FlowJo V10 (FlowJo LLC) based on gates set by the DEAB negative control, and were kept consistent for all samples analyzed.

Cell cycle analysis was done by DNA staining with propidium iodide (PI), and analyzed using FlowJo V10 cell cycle analysis tool. Briefly, cells were treated for 24 h with vehicle or Lunasin, harvested by trypsinization, counted, and assessed for viability by trypan blue exclusion. 1×10^6^ cells were washed in PBS, and resuspended in 200 μL ice-cold PBS. The cell suspension was fixed by slowly adding the suspension drop by drop to 70% ethanol for rapid dispersion, and fixed overnight at -20°C. Cells were centrifuged, and the cell pellet was resuspended in a PI Master Mix (40 μg/ mL PI, 100 μg/ mL RNase, in PBS) at a density of 1×10^6^ cells/ mL. Cells were incubated at 37°C for 30 minutes and subsequently analyzed using a FACS Calibur (BD Biosciences).

### Transwell *in vitro* invasion assay

A375 and B16-F10 cells were plated in 6-well culture plates at a density of 1×10^5^ cells per well in 2 mL of DMEM culture media. After 4 hours, the media was removed and replaced with media containing vehicle or 100 μM Lunasin for 24 h. A 24 h treatment period was selected based on our previous studies [34, 35, 37] and our observation that Lunasin reduced the number of B16-F10 ALDH^high^ cells within 24 h (Figure 3C and 3D). After treatment, cells were washed once with PBS, and harvested with TrypLE dissociation media (Gibco). Cells were counted and viability was assessed by trypan blue exclusion assay; > 95% viability was observed for all samples. Cells were replated at a density of 1×10^5^ viable cells in serum-free DMEM culture media containing vehicle or Lunasin into a transwell Boyden chamber (pore size = 8 μm) coated with Matrigel basement membrane (Corning). The bottom chamber was filled with DMEM culture media containing 10% FBS to promote invasion from the top chamber. After 24 h at 37°C, cells were removed from the top chamber by using a cotton-tipped swab, and cells adhered on the bottom layer of the insert were fixed in 100% methanol and stained in a 1% Toluidine Blue in 1% borax solution. After several washes in distilled water, membranes were allowed to air dry, and mounted onto slides with immersion oil under a 60 mm cover slip. A total of 5 fields per insert were counted and averaged to obtain the average number of cells per field.

### Murine model of experimental metastasis

All mice were handled in accordance with the Association for Assessment and Accreditation of Laboratory Animals Care international guidelines with the approval of the appropriate Institutional Animal Care and Use Committees at Indiana University, Bloomington (Protocol # 14–019–4). 2.5 × 10^5^ B16-F10 cells suspended in 100 μL phosphate buffered saline (PBS) were injected intravenously (IV) into 20 4-6 week old, female C57Bl/6 mice (Harlan) via the lateral tail vein. Mice were randomly assigned to either the control or experimental group (10 mice/group) after receiving the initial implantation of B16-F10 cells. Immediately following transplantation of melanoma cells, mice were dosed with Lunasin (30 mg / kg) or vehicle by intraperitoneal (IP) injection. The 30 mg/kg dose was selected based on our previous *in vivo* studies of human and mouse melanoma subcutaneous tumor growth [37, 56]. Mice received daily IP injections of Lunasin or vehicle until the end of the experiment 18 days post-transplantation of cells; preliminary studies demonstrated that this protocol generated numerous large lesions 22 days after injection. Upon sacrificing the mice, lungs were resected and imaged using a Leica M205 Stereoscope (Leica). Tissues were fixed in 10% formalin for 72 hours and processed for subsequent histological staining.

### Histology

After fixation in 10% formalin, lungs were transferred to 70% ethanol and stored overnight at room temperature. Tissues were dehydrated through a series of graded alcohols, and infiltrated with paraffin (Electron Microscopy Sciences). Tissues were embedded in paraffin and sectioned (thickness = 7 μm) on a microtome. Sections were transferred to SuperFrost Plus slides (Fisher) and allowed to dry overnight on a slide warmer (Fisher). Paraffin removal was initiated by several washed in xylene, and followed by rehydration of the tissues in a series of graded alcohols. Tissues were stained in hematoxylin and eosin (H&E) solutions followed by a clearing solution of xylene. After staining, Permount^TM^ mounting medium (Fisher) was applied to each slide and covered with a 60 mm cover slip (Fisher). Slides were allowed to dry at room temperature overnight and then placed in a drying oven until completely dry. Images of H&E stained slides were taken using a Leica M205 Stereoscope (Leica) as well as an EVOS light microscope (Life Technologies). Macrometastases were counted under 4.32x magnification on the Leica M205 Stereoscope. Micrometastases were counted from H&E stained non-sequential sections (n = 5) from each tissue sample using an EVOS light microscope. Images were subsequently analyzed for total tumor area using ImageJ software (NIH).

### Immunoblot analysis

Cultured cells were treated with PB or Lunasin (100 μM) for 24 hours. Acid extraction of histones was performed as described [79]. 10 μg of total purified histones were loaded per lane and run on 15% gels (Lonza). Cells were harvested and re-suspended in RIPA buffer (Sigma). Protein concentrations of cell lysates were determined by a bicinchoninic acid assay (Thermo Fisher Scientific) and 20–60 μg of total protein was loaded per lane on 10% gels (BioRad), subjected to SDS-PAGE, and transferred to a PVDF membrane (EMD Millipore). Lysates were probed with antibodies that recognize phosphorylated AKT (Cell Signaling #9916S), phosphorylated FAK (Cell Signaling #9330S), phosphorylated ERK 1/2 (Cell Signaling #4094), β-Actin (Santa Cruz #sc-47778), H3K9 (EMD Millipore #07-352), H4K12 (EMD Millipore #04-119), H4K8 (EMD Millipore #07-328), H3K14 (EMD Millipore #07-353). Densitometry and image analysis were performed using a ChemiDoc station equipped with ImageLab software (BioRad).

### Statistical analysis

GraphPad Prism 5.0 software (GraphPad Prism Software, Inc.) was used for all statistical analyses. For all *in vitro* studies, two-group comparisons between control and test samples were done by two-tailed student's t-tests and represent data from three independent experiments. Experimental metastasis data were analyzed for significance using two-tailed student's t-tests. For all tests, statistical significance was assumed when *p* < 0.05.

## SUPPLEMENTARY MATERIALS FIGURES


