# Supplementary material for: The soy-derived peptide Lunasin inhibits invasive potential of melanoma initiating cells
Source: Oncotarget. 2017 Mar 10;8(15):25525–41. doi: 10.18632/oncotarget.16066 (PMC5421948; doi:10.18632/oncotarget.16066)
Supplement: Supplementary file 1 [file oncotarget-08-25525-s001.pdf]

The soy-derived peptide Lunasin inhibits invasive potential of melanoma initiating cells

SUPPLEMENTARY MATERIALS

SUPPLEMENTARY FIGURES

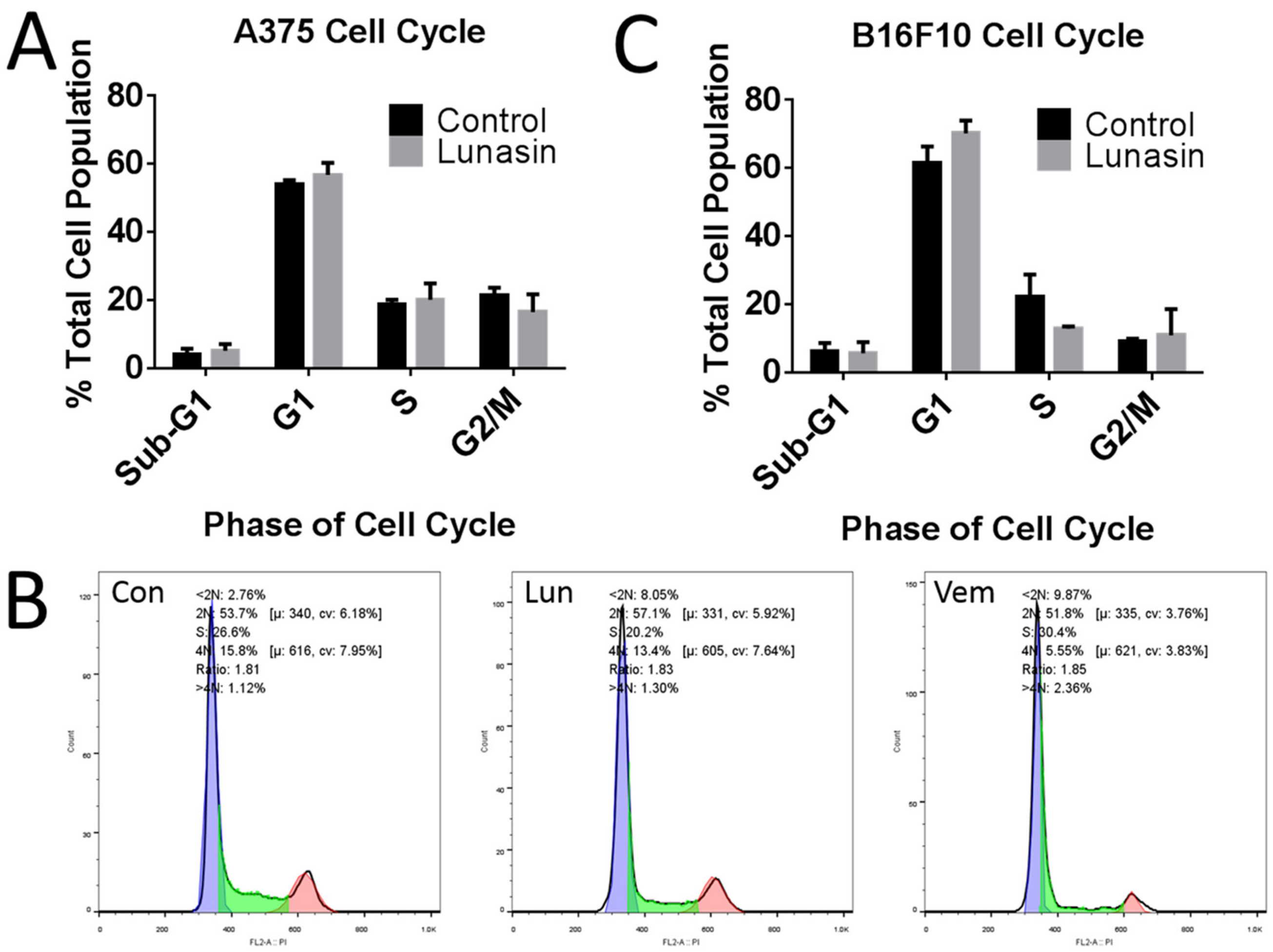

Supplementary Figure 1: Cell cycle was not significantly affected by Lunasin. A375 **(A)** and B16-F10 **(B)** ALDH^{high} cells were treated with 100 μM Lunasin, and labeled with PI for cell cycle analysis. No significant difference in populations in G1, S-phase, or G2/M was observed with Lunasin treatment compared to control in either cell line, although there was a reproducible trend toward reduced numbers of cells in S phase and an accumulation of cells in G1. A representative image shows the cell cycle curve for control, Lunasin, and vemurafenib (positive control) as analyzed using FlowJo cell cycle analysis software. Data were obtained from three independent experiments and shown as mean ± s.d.

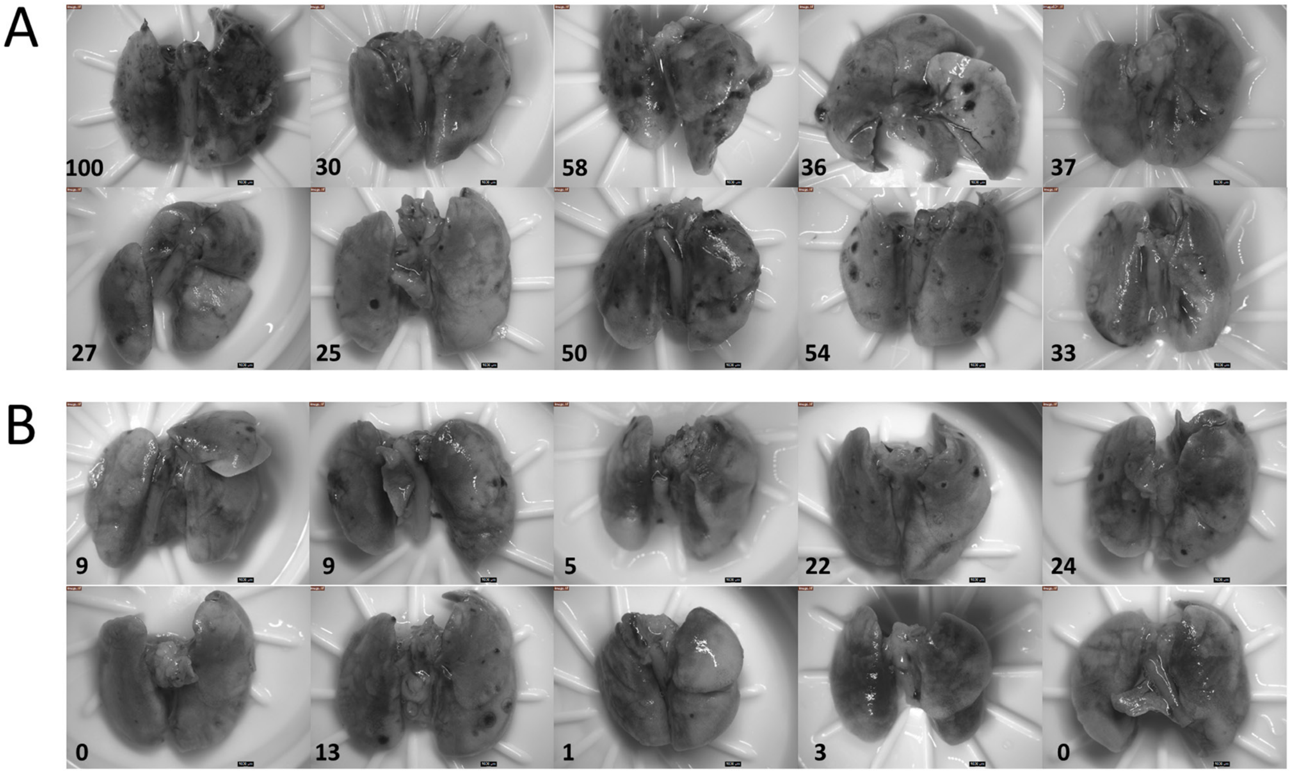

Supplementary Figure 2: Lungs resected from experimental metastasis *in vivo* model. Lungs were resected from mice receiving Lunasin or vehicle treatment after implantation of B16-F10 cells. Lunasin-treated mice displayed significantly less tumor burden in pulmonary tissues when compared to vehicle-treated mice. The number of macrometastatic lesions observed for each set of lungs is shown in the bottom left of each image.

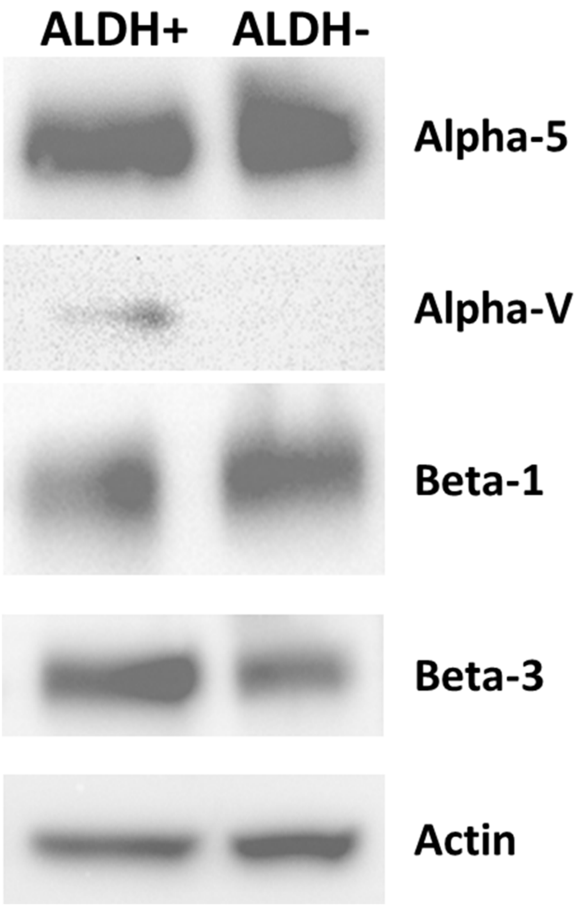

Supplementary Figure 3: Differential expression of integrin subunits by melanoma CICs. A375 ALDH^{low} and ALDH^{high} cells were isolated by FACS, and lysates were subjected to SDS-PAGE. Integrin expression profiles show that ALDH^{high} cells express higher levels of α_V and β_3 subunits when compared to ALDH^{low} cells. ALDH^{low} cells showed higher abundance of integrin α_5 and β_1 subunits when compared to samples from ALDH^{high} cells.

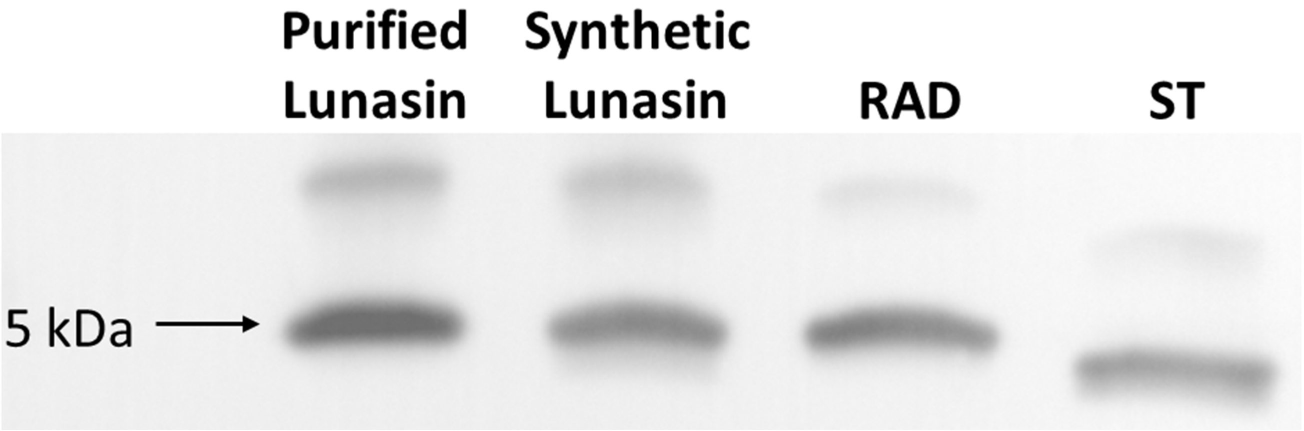

Supplementary Figure 4: Validation of Lunasin antibody recognizing mutated peptides. 200 ng of total peptide were separated on 15% polyacrylamide gels and probed against Lunasin using a rabbit polyclonal antibody (used in Figure 2 and Figure 8). The antibody detected both the mutant peptides with signal intensity similar to the native Lunasin peptide. This confirms that our antibody recognizes the mutated peptide sequences, and therefore, are suitable for the immunolocalization studies of mutant peptides.

Peptide Name (Abbreviated)	Full Amino Acid Sequence
Purified Lunasin	SKWQHQQDSCRKQLQGVNLTPCEKHIMEKIQGRGDDDDDDDDDN
Synthetic Lunasin	SKWQHQQDSCRKQLQGVNLTPCEKHIMEKIQGRGDDDDDDDDD
RAD (RGD-mutated)	SKWQHQQDSCRKQLQGVNLTPCEKHIMEKIQGRADDDDDDDDD
ST (Scrambled Tail)	SKWQHQQDSCRKQLQGVNLTPCEKHIMEKIQGRGDAGGKVGVA

Supplementary Figure 5: Sequences of synthesized peptides. Peptides based on Lunasin's initially published 43 amino acid sequence were synthesized by China Peptides to represent altered functional domains. Mutated amino acid sequences for each peptide are underlined in red. Purified Lunasin was used in all experimental conditions, and varies from the initially reported Lunasin sequence by a single asparagine residue on the C-terminal end. The native purified Lunasin has activity equivalent with the synthetic 43-amino acid Lunasin sequence ([35], data not shown).
